# Supplementary material for: Diagnostic and treatment delay in extrapulmonary tuberculosis and association with mortality: Experiences from Mbeya, Tanzania
Source: PLoS One. 2025 Mar 25;20(3):e0320691. doi: 10.1371/journal.pone.0320691 (PMC11936283; doi:10.1371/journal.pone.0320691)
Supplement: S1 File — (PDF) [file pone.0320691.s001.pdf]

S1. Classification of comorbidities.

Classification of comorbidities :

Hematological disease:
1)Pancytopenia
7) (Hypochromic microcytic) anemia.
12) Anemia - Hb not stated, but tranfused
26) Leucocytosis -result not noted
28) Blood smear with atypical cells
41) Anemia stated, Hb not stated, not transfused
60) Neutropenia
64) Massive lymphocytosis
67) Sickle Cell Disease
76) Myelodysplastic syndrome
28- Suspected hematological malignancy

GI disease (excluding liver disease)

21) Pos. H pylori test
25) Melena
37) Bloody stools
38) Upper GI bleeding
47) Excessive diarrhoea

Liver disease

2) Increased ALT/AST and/OR bilirubin
3) Liver abscess on USS - not drained
5) Suspected liver cirrhosis secondary to hepatitis or schistosomiasis (no test results reported to support this)
22) Jaundice of unknown reason
40) Suspected spontaneous bacterial peritonitis
42) Liver cirrhosis
43) Fatty liver
75) Chylous ascites
12- Suspected liver failure due to schistosomiasis
17- (+/- suspected) Liver cirrhosis
31 - Suspected liver failure
37- Suspected spontaenous bacterial peritonitis
40- Suspected hepatorenal syndrome

Heavy alcohol consumption
14) Heavy alcohol consumption

Malignancy (confirmed, suspected and previous malignancy)
15) Kaposis sarkoma (clinical or verified)
28) Blood smear with atypical cells
49) Previously treated for lymphoma
61) Started chemotherapy
2 - Lymphoma
5 - colonic cancer
16 - Suspected cancer of the pancreas
18- Renal cancer
21- Hepatocellular carcinoma
28- Suspected hematological malignancy
29 - Sarcoma
30 - Supected malignancy
24- Suspected Kaposis sarcoma
41- Breast cancer

Renal disease -
9) Post-renal kidney failure.
27) Increased Creatinine
32)Known renal failure
78) Nephrotic syndrome
80) Sups. acute kidney failure
19- Suspected nephrotic syndrome
39 - Chronic kidney failure

Cardiovascular disease
13) Stroke
39) Suspected/known CHF
45) Hypertension
54) Suspected peripartum cardiomyopathy
71) Rheumatic heart disease
8- Suspected CHF due to R. atrial myxoma
9 - Suspected cardiomyopathy due to HIV
11- Suspected CHF

Diabetes Mellitus
46) DM 2
63) Diabetic foot

Malnutrition
65) Moderate Acute Malnutrition
66) Severe acute malnutrition
86) Relapse of SAM

Other infectious conditions
6) previously treated for neck abscess
16)Oral thrush
20) Positive Hep B bloodtest
23) Perineal abscess
30) Susp. bacterial empyema
33) Previously treated for schistosomiasis
36) Treated for Syphilis (VDRL pos)
40) Suspected spontaneous bacterial peritonitis
44) Septicemia
48) Treated for typhoid
50) Tonsilitis
51) Oesophagal candiasis
56) Suspected cryptococcal meningitis
70) Scabies
81) Severe malaria
1 - Pneumonia w/ suspected empyema
3 - Suspected empyema
6- Mastitis
27 - Scrotal abscess
34 - Infected epidermal cyst
36 - Pneumonia
37- Suspected spontaenous bacterial peritonitis

Trauma -
8) Stabbed in L side of chest some months ago, received chest tube
52) Caesarioan sectio (<4 weeks ago)
53) Post delivery (<4 weeks ago)
59) Suffer trauma during road traffic accident (<4 weeks ago)
42 - Post traumatic pleural effusion

TB complications:
29) Given full course of ATT on suspicion of EPTB without improvement
35) Treated for TB twice
55) Suspected pulmonary TB
57) On ATT for suspected pulmonary TB without any evidence for the diagnosis
68) Received post exposure TB prophylaxis

87) Restarted ATT due to treatment interruption

HIV complications
4) Suspected treatment failure of ARVs, ARV regime changed
10) Suspected treatment failure/interruption of ARVs
15) Kaposis sarkoma (clinical or verified)
16)Oral thrush
19) Suspected HIV, but dies before testing
31) Suspected IRIS
34) Changed ARVs due to lipodystrophy
51) Oesophagal candiasis
56) Suspected cryptococcal meningitis
69) Post delivery ARVs as mother is HIV+
74) Third line ARVs
79) Mother newly diagnosed with HIV
83) Mother known HIV positive
84) Mother HIV pos, did not receive ARVs post delivery
9 - Suspected cardiomyopathy due to HIV
10 - AIDS with unknown cause of death
13 - Reactive follicular hyperplasia in HIV patient
24- Suspected Kaposis sarcoma
26- Chronic unspecific lymphadenopaty in HIV+ patient
33 - Lipodystrophy due to ARVs

Other
11) Breastfeeding
17) Works in mining industry
18) Ovarian cyst
24) Inguinal lymphadenopathy
58) Albumin below 20 g/dl
62) Potassium >6mmol/l
72) Hydrocehphalus
73) Received VP shunt
77) Comatose
82) Recent dogbite
85) Decorticate posturing
