# Supplementary material for: Diagnostic and treatment delay in extrapulmonary tuberculosis and association with mortality: Experiences from Mbeya, Tanzania
Source: PLoS One. 2025 Mar 25;20(3):e0320691. doi: 10.1371/journal.pone.0320691 (PMC11936283; doi:10.1371/journal.pone.0320691)
Supplement: Attachment — Submitted filename: Rebuttal_letter_auresp_2.docx [file pone.0320691.s005.docx]

Rebuttal letter – *PLoS One*	20.02.2025

Dear Sirs,

Thank You for your time and consideration in reviewing our manuscript (ONE-D-24-41844R1) titled "Diagnostic and treatment delay in extrapulmonary tuberculosis and association with mortality: experiences from Mbeya, Tanzania."

We appreciate the valuable feedback provided by the reviewers and the editorial team. In this revised version, we have addressed all the points raised and made the necessary modifications to enhance the clarity, accuracy, and readability of our manuscript.

Below, we provide a point-by-point response to the comments from the editor and reviewers:

**Editor’s Comments:**

1. **Proofreading:**
   - A thorough proofreading of the manuscript has been conducted to correct typographical, grammatical, and clarity issues.
2. **Line 72: EPTB abbreviation**
   - The full form "Extrapulmonary Tuberculosis (EPTB)" has been written out at the beginning of the sentence.
3. **Line 181: Description of Fig 1 should include person, place, and time.**
   - The following descriptions have been added to ensure clarity:
     - Patient delay: The time (days) from the patient’s report of symptom onset to the first visit to a healthcare provider.
     - Healthcare system diagnostic delay: The time from the first healthcare visit to diagnostic sampling for suspected EPTB.
     - Total diagnostic delay: The sum of patient delay and healthcare system diagnostic delay.
     - Delay in ATT initiation: The time from diagnostic sampling to the start of anti-tuberculosis treatment.
     - Total treatment delay: The time from symptom onset to the start of ATT.
4. **Line 201: Correction to "Statistical analysis"**
   - Apologies for the oversight. The correction has been made as per the suggestion.
5. **Reference Check for Retractions:**
   - The citation of the Global Tuberculosis Report has been updated to the 2024 edition.
   - A comprehensive review of the reference list was conducted. No cited articles were found to be retracted. A manual search in PubMed and an AI-based search for retraction notifications confirmed this.

**Reviewer #1’s Comments:**

1. **Structuring Population and Study Design Section:**
   - The description of the study design and population has been restructured under subheadings (e.g., Exclusion criteria, Data sources) to improve readability.
   - The data source for the study is now clearly stated within this section.
2. **Table 2: Clarification on Referral Categories:**
   - The visual structure of Table 2 has been revised to clearly indicate that there are four referral categories. Additional table lines were added to avoid confusion regarding the number of categories.

**Reviewer #3’s Comments:**

- Reviewer #3 has indicated that all comments have been satisfactorily addressed, and no further revisions were required.

We sincerely appreciate the time and effort taken by the reviewers and editorial team to provide constructive feedback. We believe that the revisions have strengthened the manuscript and made it suitable for publication in PLOS ONE.

Thank you for your consideration. We look forward to your feedback.

Best regards,

Erlend Grønningen

MD, PhD, DTMH
